# Self-rated health and smoking among physicians and general population with higher education in Estonia: results from cross-sectional studies in 2002 and 2014

**DOI:** 10.1186/s13690-019-0376-7

**Published:** 2019-11-25

**Authors:** Kersti Pärna, Inge Ringmets, Sävelin Siida

**Affiliations:** Institute of Family Medicine and Public Health, University of Tartu, Ravila 19, 50411 Tartu, Estonia

**Keywords:** Self-rated health, Smoking, Physicians, General population with higher education, Sociodemographic factors, Estonia

## Abstract

**Background:**

Poor self-rated health (SRH) and smoking have consistently been shown to be related to mortality. The aim of this study was to explore SRH and smoking among physicians and general population with higher education in Estonia in 2002 and 2014 and to analyse the association of SRH with smoking and sociodemographic factors.

**Methods:**

This study was based on cross-sectional postal surveys among physicians and general population with higher education in Estonia n in 2002 and 2014. Calculation of age-standardized prevalence of SRH and current smoking with 95% confidence intervals (CI) was performed. Multivariate logistic regression analysis was used to measure association between SRH (at-least-good vs less-than good) and smoking status, study year, age group, ethnicity, and marital status. Fully adjusted odds ratios (OR) with 95% CI were computed.

**Results:**

Age-standardized prevalence of at-least-good SRH was 71.3 and 80.6% among male physicians, 68.4 and 83.1% among female physicians, 45.4 and 67.4% among men with higher education, and 44.7 and 63.1% among women with higher education in 2002 and 2014, respectively. Age-standardized prevalence of current smoking was 26.0 and 15.6% among male physicians, 10.2 and 5.9% among female physicians, 38.7 and 22.2% among men with higher education, and 20.9 and 16.4% among women with higher education in 2002 and 2014, respectively. There was no significant gender difference in at-least-good SRH, but prevalence of current smoking was significantly higher among men in both study groups in 2002 and 2014. Compared to year 2002, odds to have at-least-good SRH was higher in 2014 (OR = 1.64; 95% CI 1.16–2.31 among male and OR = 2.36; 95% CI 2.02–2.75 among female physicians, OR = 1.49; 95% CI 1.07–2.07 among men and OR = 2.40; 95% CI 1.84–3.13). Odds to have at-least-good SRH was significantly higher among non-smokers (except female physicians), in the youngest age group, and among Estonians.

**Conclusions:**

This study gave an overview of differences in SRH and smoking between two target groups with higher education in two timepoints highlighting the importance of addressing smoking cessation counselling and health promotion campaigns in the population by different subgroups in Estonia.

## Background

Poor self-rated health (SRH) and smoking have consistently been shown to be related to future mortality in different countries in Europe [1–3]. According to this, poorer SRH could be associated with smoking.

Poor SRH has decreased during the last decades among adult general population in European countries and has been more prevalent among lower socioeconomic class [4–6]. However, the number of published articles concerning SRH among physicians is much lower. In Lithuania, 70.5% of male and 58.9% of female physicians rated their health as at-least-good in 2006 [7]. In Norway 88.3% of male and 88.1% of female physicians, in Germany 62.9% of male and 64.1% of female physicians rated their health as at-least-good in 2008 [8].

Similarly to poor SRH, current tobacco smoking has decreased among adults in developed countries in Europe [9, 10] with higher prevalence among lower socioeconomic class in accordance to tobacco epidemic model [11]. Current tobacco smoking has also decreased among physicians in high income countries worldwide (e.g United States, Australia, Scandinavian countries) [12, 13] being comparable with the ‘mature’ smoking epidemic among physicians [14] with lower prevalence of smoking among physicians than among general population within the country. In Estonia there is studied SRH [15, 16] and smoking [17, 18] by socioeconomic position among general population and smoking among physicians [19], but there is no studies concerning SRH among physicians. Moreover, whether the association of SRH and smoking differ among physicians and general population with higher education, is not investigated in Estonia.

The aim of this study was to explore SRH and smoking among physicians and general population with higher education in Estonia in 2002 and 2014 and to analyse association of SRH with smoking and sociodemographic factors.

## Methods

### Study population and design

The present study was based on aggregated data of cross-sectional postal smoking surveys among physicians [20, 21] and health behaviour surveys among adult population in Estonia in 2002 and 2014 [22, 23].

### Surveys among physicians in 2002 and 2014

The methods used in two cross-sectional postal surveys among physicians have been extensively described elsewhere [20, 21]. In short, all practicing physicians who were permanent residents of Estonia were eligible for sampling in 2002 (*n* = 4140) and 2014 (*n* = 5666). In 2002, all practicing physicians were identified from the database of Estonian Health Insurance Fund and questionnaires were mailed to the workplace address of the physicians. In 2014, the sample of all practicing physicians was based on data from the Estonian Health Care Professionals Registry and questionnaires were mailed to the home address of physicians. To receive home addresses, data from the Estonian Health Care Professionals Registry were linked with the Population Registry in Estonia. The number of respondents was 2747 in 2002 and 2903 in 2014. The crude response rates were 66.3, and 52.0%, respectively. The corrected response rates (excluding the physicians who were unavailable, retired, had an incorrect address, had left Estonia or haddied) were 67.8% in 2002 and 53.1% in 2014. Similar self-administered questionnaires were used to collect information on SRH, smoking and sociodemographic factors in 2002 and 2014.

The target group of the present study were 25–64-years-old physicians in 2002 (*n* = 2521) and in 2014 (*n* = 2303) who answered to the questions concerning SRH and smoking status (Table 1).

**Table 1:** Sample of 25–64-years old Estonian physicians and general population with higher education, 2002 and 2014

Study year	Physicians			General population with higher education		
	Men	Women	Total	Men	Women	Total
2002	415	2106	2521	62	128	190
2014	403	1900	2303	245	472	717
Total	818	4006	4824	307	600	907

### Surveys among general population in 2002 and 2014

The study was based on data drawn from two cross-sectional postal surveys of Health Behaviour among Estonian Adult Population conducted among 16–64-year-old adults in every second year since 1990. A random sample, stratified by age, gender and place of residence, of the Estonian population aged 16–64 was taken from the Population Registry. The methodology of survey of Health Behaviour among Estonian Adult Population is described in more detail elsewhere [22, 23]. In 2002 the initial sample consisted of 2000 adults, in 2014 the sample size was 5000 adults. The crude response rate of the inital sample was 66.9% in 2002 and 51.5% in 2014. The methodology and the questionnaires used in the surveys were harmonized to provide comparability between study years. Corrected response rate (exluding those, who did not live at the address provided, no letter box available, not living in Estonia, had died) was not available for 2002, but 53.5% in 2014.

The target group of the present study was 25–64-years-old general population with higher education in 2002 (*n* = 190) and in 2014 (*n* = 717) who answered to the questions concerning SRH and smoking status (Table 1).

### Study variables

**SRH** was measured by a single question concerning self-assessment of the current state of health. Five options of responses were dichotomized to at-least-good (good, rather good) and less-than good (average, rather poor, poor) SRH.

**Smoking status** was determined by combining answers to several questions concerning daily (smoking at least one cigarette a day), occasional (smoking less than one cigarette a day, past and never smoking and was classified as current (smoking at least one cigarette or less than one cigarette a day), past (not currently smoking) and never (smoked less than 100 cigarettes during her/his life) smoking.

**Study groups** were 25–64-years-old physicians and general population with higher education in Estonia. Population with higher education was defined by the highest completed educational level which included at least 15 years of studies.

**Study years** were 2002 and 2014 for both study groups.

**Age** was measured in full years and analysed in four age groups: 25–34, 35–44, 45–54, 55–64.

**Ethnicity** referred to self-determined national identity and was analysed in two groups: Estonians and non-Estonian (mainly Russians).

**Marital status** was classified into three groups: married or cohabiting, single, separated or divorced or widowed.

### Statistical analysis

As gender was associated with SRH and smoking, the results were analysed separately for males and females. The primary data analysis involved the calculation of age-standardized prevalence of SRH and current smoking using European standard population [24] with 95% confidence intervals (CI). Multivariate logistic regression analysis was used to measure the association between SRH (at-least-good vs less-than good) and smoking status, study year, and sociodemographic factors (age group, ethnicity, and marital status) among physicians and general population with higher education. Fully adjusted odds ratios (OR) and the corresponding 95% CI were computed.

The data was analysed using the statistical package Stata 12.1 [25].

## Results

**Fig. 1:**
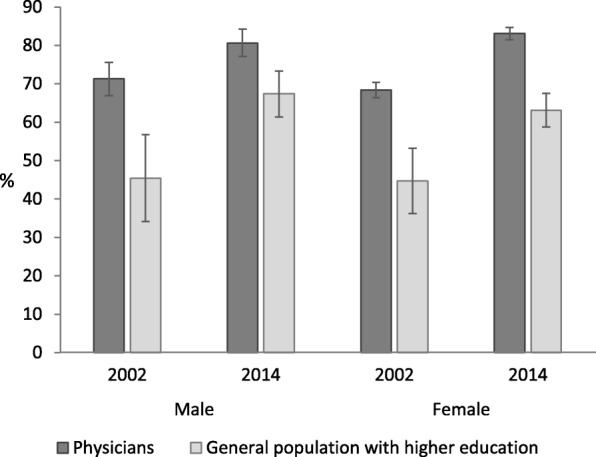
Age-standardized prevalence of at-least-good SRH among physicians and general population with higher education in 2002 and 2014 in Estonia. Data shown as age-standardized prevalence of at-least-good SRH with 95% confidence intervals (CI). Age standardization was performed using the European standard population [24]

Age-standardized prevalence of at-least-good SRH among male physicians was 71.3% (95% CI 66.9–75.6) in 2002 and 80.6% (95% CI 77.1–84.2) in 2014 and among female physicians 68.4% (95% CI 66.4–70.4) and 83.1% (95% CI 81.5–84.7), respectively (Fig. 1). Age-standardized prevalence of at-least-good SRH among men with higher education was 45.4% (95% CI 34.1–56.7) in 2002 and 67.4% (95% CI 61.4–73.3) in 2014, among women with higher education 44.7% (36.2–53.2) and 63.1% (95% CI 58.7–67.5), respectively.

**Fig. 2:**
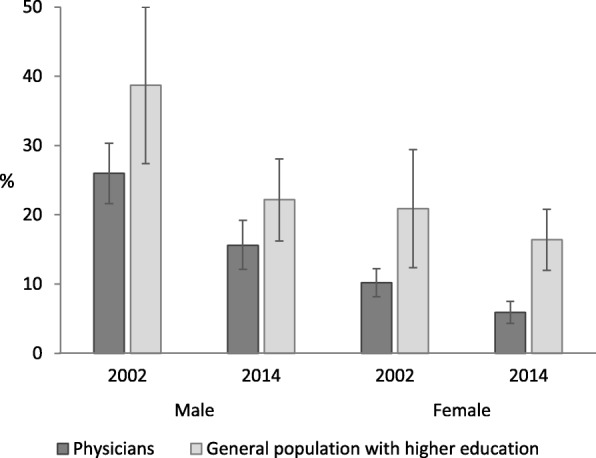
Age-standardized prevalence of current smoking among physicians and general population with higher education in 2002 and 2014 in Estonia. Data shown as age-standardized prevalence of current smoking with 95% confidence intervals (CI). Age standardization was performed using the European standard population [24]

Age-standardized prevalence of current smoking among male physicians was 26.0% (95% CI 21.4–30.5) in 2002 and 15.6% (95% CI 12.0–19.2) in 2014 and among female physicians 10.2% (95% CI 8.9–11.6) and 5.9% (95% CI 4.9–7.0), respectively (Fig. 2). Age-standardized prevalence of current smoking among men with higher education was 38.7% (95% CI 27.0–50.5) in 2002 and 22.2% (95% CI 17.0–27.4) in 2014 and among women with higher education 20.9% (95% CI 13.6–28.3) and 16.4% (95% CI 13.0–19.9), respectively.

In fully adjusted logistic regression model SRH was associated with smoking status (except among female physicians), study year, age, and ethnicity among physicians as well as among general population with higher education. No association was found between at-least-good SRH and marital status in both study groups.

Compared to currently smoking male physicians, odds to have at-least- good SRH was significantly higher among past and never smoking male physicians (Table 2). No association was found between SRH and smoking among female physicians. Compared to currently smoking men and women with higher education, odds to have at-least-good SRH was significantly higher among never smoking men and women with higher education. Compared to study year 2002, odds to have at-least-good SRH was significanly higher among men and women in both study groups in 2014. Compared to the youngest age group, odds to have at-least-good SRH was significantly lower in older age groups in both study groups. Compared to non-Estonians, odds to have at-least-good SRH was significantly higher among Estonian men and women in both study groups.

**Table 2:** Odds ratios (OR) for SRH (at-least-good vs less-than-good) and 95% confidence intervals (CI) among physicians and general population with higher education by gender in 2002 and 2014

Characteristic	Physicians		Population with higher education	
	Adjusted^a^ OR (95% CI)		Adjusted^a^ OR (95% CI)	
	Men	Women	Men	Women
Smoking status				
Current	1	1	1	1
Past	1.85 (1.20–2.85)	1.07 (0.80–1.45)	1.20 (0.90–1.59)	1.32 (0.99–1.78)
Never	2.39 (1.58–3.60)	1.04 (0.80–1.34)	1.65 (1.21–2.24)	1.39 (1.08–1.79)
Study year				
2002	1	1	1	1
2014	1.64 (1.16–2.31)	2.36 (2.02–2.75)	1.49 (1.07–2.07)	2.40 (1.84–3.13)
Age group				
25–34	1	1	1	1
35–44	0.65 (0.35–1.22)	0.49 (0.37–0.66)	0.51 (0.37–0.71)	0.65 (0.48–0.87)
45–54	0.36 (0.20–0.67)	0.28 (0.21–0.38)	0.27 (0.19–0.38)	0.35 (0.26–0.47)
55–64	0.19 (0.10–0.35)	0.16 (0.12–0.21)	0.15 (0.10–0.22)	0.16 (0.12–0.22)
Ethnicity				
Non-Estonian	1	1	1	1
Estonian	1.50 (1.02–2.21)	1.96 (1.62–2.37)	1.66 (1.25–2.22)	2.44 (1.94–3.06)
Marital status				
Married/cohabiting	1	1	1	1
Single	0.80 (0.40–1.59)	0.83 (0.66–1.06)	1.28 (0.93–1.76)	1.04 (0.79–1.37)
Divorced/widowed	0.91 (0.44–1.85)	0.88 (0.73–1.07)	0.67 (0.40–1.12)	0.96 (0.70–1.31)

^a^Each OR was adjusted for all the other variables in the table

## Discussion

This study focused on SRH and smoking among physicians and general population with higher education in Estonia in 2002 and 2014, and analysed association of SRH with smoking and sociodemographic factors. The main findings from the study were, *first*, that compared to general population with higher education, prevalence of at-least-good SRH was higher and prevalence of current smoking lower among physicians. *Second*, compared to 2002, prevalence of at-least-good SRH was higher and prevalence of current smoking lower in 2014 in both study groups. *Third*, there was no significant gender difference in at-least-good SRH, but prevalence of current smoking was significantly higher among men in both study groups. *Fourth,* non-smokers (except female physicians), adults in the youngest age groups and Estonians considered their health better.

### Strengths and limitations

The surveys of smoking among physicians and of health behaviour among adult general population present an outstanding opportunity to analyse SRH and smoking status among physicians and general population with higher education in 2002 and 2014 in Estonia because of the similar study design (postal cross-sectional surveys) and methodology (representative samples).

Before discussing the results some limitations of this study should be addressed. *First*, response rates in surveys among physicians as well as among general population was much lower in 2014 than in 2002, as is the case with postal surveys in most populations worldwide [26]. The non-respondents may have revealed different patterns of SRH and smoking status compared with those who returned the questionnaire. Thus, possible weaker participation of adults with lower SRH and smoking behaviour has to be considered [27, 28]. *Second*, smaller initial sample size in health behaviour survey among general population in 2002 might decrease power of detection of significant differences between study groups. *Third*, dichotomizing of SRH as at-least-good and less-than-good was done assuming that respondents who rate their health as average are feeling not healthy. SRH refers to subjective health assessment and therefore there is not possible to draw a strict line between at-least-good and less-than-good health. Such a categorization can affect the results as it is not definite to which group the ‘average’ is more similar to [29], however, a sensitivity analysis using different categorization for SRH in earlier study demonstrated similar associations with sociodemographic and -economic factors. Also, it has been reported that whether SRH was categorized to two groups or analyzed based on five point scale, the results were similar [30]. *Fourth*, self-reports of smoking tends to slightly underestimate the actual amount of smoking, especially among physicians as they know more about the devastating effects of smoking than the general population despite these caveats, several inferences can be drawn.

#### SRH

Compared to general population with higher education prevalence of at-least-good SRH was significantly higher among physicians of both genders in both study years in Estonia. This means that physicians’ better knowledge concerning illnesses and increased prevalence of common mental disorders reported among physicians worldwide [31–33] were not associated with rating their health poorer. On the contrary, physicians rated their health better than general population with higher education. The age standardized prevalence of at-least-good SRH was significantly higher in 2014 than in 2002 among physicians as well as among general population with higher education in Estonia. While about two third to three fourth of physicians rated their health at-least-good in 2002, then more than four fifth agreed with this choice in 2014 in Estonia. At the same time, in 2002, less than half, but in 2014 about two third of general population with higher education rated their health at-least good in Estonia. Similar increase of prevalence of at-least-good health was described in earlier studies among general adult population in Estonia [15] and in Eastern European countries [34]. There was not found significant gender differences in prevalence of at-least good SRH among physicians and general population with higher education, which confirms the findings of earlier study in Estonia [15].

#### Current smoking

Compared to general population with higher education, prevalence of current smoking was significantly lower among physicians of both genders in both study years in Estonia. Previous study [19] in Estonia indicated that prevalence of smoking among physicians was much lower than that observed in the general population demonstrating that Estonia is comparable with ‘mature’ smoking epidemic among physicians [14]. The findings of current study indicated that physicians are far less likely to smoke than general population with higher education. The age standardized prevalence of current smoking was significantly lower in 2014 than in 2002 among male and female physians as well as among men and women in general population with higher education in Estonia. Similar decrease of prevalence of current smoking was described in earlier studies in Estonia [17] and in other developed European countries [35, 36]. The finding of this study that the prevalence of current smoking was significantly higher among men than women in both study groups in both study years is consistent with that seen in earlier studies in Estonia [17–20]. Moreover, notably higher smoking prevalence among men is common in the former Soviet countries [37], meanwhile in the most Scandinavian and Western European countries gender difference in current smoking prevalence is much smaller [38].

#### Association of SRH (at-least-good vs less-than-good) with smoking and sociodemographic factors

Fully adjusted logistic regression model confirmed similar association of at-least-good SRH with nonsmoking (except female physicians), study year (2014), the youngest age group, and Estonian ethnicity in both study groups.

The fact, that at-least-good SRH was associated with study year 2014 was in accordance with higher prevalence of at-least good SRH in the year 2014 in this study. Association between at-least-good SRH and nonsmoking was found among male physicians and among men and women of general population with higher education. The fact, that SRH was not associated with smoking status among female physicians could be explained with not intensive smoking among them. Compared to the adults in the youngest age group adults in older age groups had lower odds to assess their health as at-least-good in Estonia. This finding was in accordance with the results from previous studies [16, 34]. Compared to non-Estonian men and women, odds to have at-least-good SRH was significantly higher among Estonians in both study groups. In earlier studies describing data from Estonia, controversial results were reported. Some studies showed that compared to non-Estonians, Estonians were more likely to rate their health as good [39, 40], but some studies found associations between SRH and nationality only among women [15, 30].

## Conclusions

This paper provided a new information concerning SRH and smoking among physicians as well as among general population with higher education in Estonia in 2002 and 2014. Compared to the general population with higher education, prevalence of at-least-good SRH was higher and of current smoking lower among physicians in both study years. Compared to 2002, prevalence of at-least-good SRH was higher and prevalence of current smoking lower in both study groups in 2014. There was no gender differences in prevalenc of at-least-good SRH, but prevalence of current smoking was higher among men compared to women in both study groups. Non-smokers (except female physicians), adults in the youngest age group and Estonians considered their health better in both study groups.

In conclusion, this study gave an overview of SRH and smoking in two target groups with higher education in two timepoints highlighting the importance of addressing smoking cessation counselling and health promotion campaigns in the population in Estonia by different subgroups.

## Data Availability

The datasets of physicians used and analysed during the current study are available from the corresponding author on reasonable request. The datasets of general population used and analysed during the current study are available from the responsible coordinators of these surveys in Estonia on reasonable request.
